# Evaluation of Olfactory and Gustatory Function of HIV Infected Women

**DOI:** 10.1155/2016/2045383

**Published:** 2016-03-07

**Authors:** Ayotunde James Fasunla, Adekunle Daniel, Ukamaka Nwankwo, Kehinde Mobolanle Kuti, Onyekwere George Nwaorgu, Olusina Olusegun Akinyinka

**Affiliations:** ^1^Department of Otorhinolaryngology, College of Medicine, University of Ibadan and University College Hospital, Ibadan 200212, Nigeria; ^2^APIN/PEPFAR Clinic, University College Hospital, Ibadan 200212, Nigeria; ^3^Department of Paediatrics, College of Medicine, University of Ibadan and University College Hospital, Ibadan 200212, Nigeria

## Abstract

*Background*. Compliance with medication requires good sense of smell and taste.* Objective*. To evaluate the olfactory and gustatory function of HIV infected women in Ibadan, Nigeria.* Methods*. A case control study of women comprising 83 HIV infected women and 79 HIV uninfected women. Subjective self-rating of taste and smell function was by visual analogue scale. Olfactory function was measured via olfactory threshold (OT), olfactory discrimination (OD), olfactory identification (OI), and TDI using “Sniffin' sticks” kits and taste function (Total Taste Strips (TTS) score) measurement was by taste strips.* Results*. The mean age of the HIV infected women was 43.67 years ± 10.72 and control was 41.48 years ± 10.99. There was no significant difference in the self-reported assessment of smell (*p* = 0.67) and taste (*p* = 0.84) of HIV infected and uninfected women. Although the mean OT, OD, OI, TDI, and TTS scores of HIV infected and uninfected women were within the normosmic and normogeusic values, the values were significantly higher in the controls (*p* < 0.05). Hyposmia was in 39.7% of subjects and 12.6% of controls while hypogeusia was in 15.7% of subjects and 1.3% of controls.* Conclusions*. Hyposmia and hypogeusia are commoner among the HIV infected women than the HIV uninfected women and the risk increases with an increased duration of highly active antiretroviral therapy.

## 1. Introduction

Women contribute significantly to socioeconomic growth and development of a society. They also play significant role in home management, family care, and upbringing of children. In most African countries, the culture demands that women prepare safe daily meal for the family and supervise intake of children's medication when they are ill. They therefore need good sense of smell and taste to prepare savory meal and ensure a safety home environment. However, disease conditions which affect taste and/or smell organs make them not safely perform these roles. The consequences include inability to identify spoilt food or detect smoke in fire or faulty electrical appliances thereby exposing family members to avoidable dangers [1]. They may even add more sugar or salt to food than desired by other members of the family in an attempt to improve the taste thereby making the food taste unpalatable [2–4].

Human immunodeficiency virus (HIV) infection is a public health problem which is more prevalent in the Sub-Sahara Africa region [5, 6]. Women are more infected with HIV than men [7, 8]. HIV infection is a systemic disease which affects virtually every organ-system of the body but with affinity or preference for nervous tissues and immune systems [9–11]. Olfactory nerve endings are located in the olfactory mucosa at the roof of the nasal cavities. Taste buds, which are located in the oral, nasal, and pharyngeal mucosae, are responsible for mediating sweet, sour, salty, and bitter taste. HIV infected individuals are susceptible to opportunistic infections of the upper aerodigestive tract [12, 13] which may affect the function of these taste buds and olfactory nerve endings [14]. The dysfunction of taste and smell can impair food intake leading to weight loss. It may also influence compliance with antiretroviral medication thereby contributing to poor therapeutic outcome [14]. These have implications on health and quality of life. Although studies have shown that dysfunction of taste and smell is a side effect of medications [15, 16], it is unclear whether antiretroviral medications affect craving or aversion for a particular type of food taste. The effect of HIV and antiretroviral medication on taste and smell of women has not been investigated in Sub-Sahara Africa. Therefore, this study was designed to evaluate the olfactory and gustatory function of HIV infected women in Ibadan, Nigeria.

## 2. Patients and Methods

### 2.1. Study Design

This was a case control study of women at University College Hospital (UCH), Ibadan, between March 2015 and June 2015. The cases were HIV infected women on highly active antiretroviral therapy (HAART) at the President's Emergency Plan for AIDS Relief (PEPFAR) Clinic, UCH, being supported by AIDS Prevention Initiative Nigeria (APIN). The controls were HIV uninfected women who are students and staff of UCH. The study was approved by the Joint University of Ibadan/University College Hospital, Ibadan (Nigeria) Ethical Review Committee. Informed consent was obtained from the participants in the study.

### 2.2. Sampling Method

The first ten HIV infected women who gave consent to participate in the study at the PEPFAR clinic on each day of the study were recruited and investigated. Women with history of smoking, rhinorrhea, nasal growth or obstruction, head injury, nasal surgeries, and pregnancy were excluded from the study. All the participants refrained from taking food or drugs orally and exposure to perfume two hours before the commencement of the tests.

### 2.3. Data Collection Procedures

#### 2.3.1. Structured Questionnaire

A structured questionnaire was used to obtain relevant information on sociodemography, nasal disease, head injury, use of perfume, perception of taste and smell, preference for sweet, salty, sour, and bitter tasting substances, duration of HIV diagnosis, type of HAART, duration of HAART use, and other clinical data from the participants. Social stratification of the participants was based on occupational strata as devised by Famuyiwa et al. [17].

#### 2.3.2. ENT Examination

Ear, nose, and throat examinations of the participants were carried out to exclude the presence of nasal pathologies like nasal discharge, polyps, or tumors and oral/throat thrush, ulcers, or lesions.

#### 2.3.3. Subjective Self-Rating of Smell and Taste Function

All the participants had initial subjective rating of their ability to perceive smell and taste on a visual analogue scale (VAS). The score for each variable ranged from 1 to 10 where 1 represented complete loss of perception of smell or taste and 10 represented excellent perception of smell or taste. A score of 1-2 is very poor, 3-4 is poor, 5-6 is good, 7-8 is very good, and 9-10 is excellent. Thereafter, all the participants in both the test and control groups had both olfaction and taste tests carried out on them.

#### 2.3.4. Virology and Immunology

The participants' Nadir CD4^+^ cell count (cells/*μ*L) and viral load (copies/mL) and their most recent CD4^+^ cell count (cells/*μ*L) and viral load (copies/mL) were retrieved from their clinic records. The protocol at the clinic included determination of the CD4^+^ cell count and plasma viral loads of HIV infected individual at six-month interval. The CD4^+^ cell count was determined using the Partec (Munster, Germany) CD4^+^ easy count based on the principle of no lysing, no wash. For the purpose of this study, HIV infected women were categorized based on the level of their CD4 cell counts (cells/*μ*L) into stage 1 (≥500 cells/*μ*L), stage 2 (200–499 cells/*μ*L), and stage 3 (<200 cells/*μ*L) in line with Centers for Disease Control and Prevention staging system [18].

#### 2.3.5. Test of Smell

The test of smell comprises odour threshold (OT), odour discrimination (OD), and odour identification (OI). These were performed using pen-like odour dispensing devices called “Sniffin' sticks.” Individual pen was positioned approximately 2 cm in front of the participants' nostrils for 3 seconds. Participants were blindfolded during the OD and OT tests to avoid visual identification of the pens.

*Odour Identification Testing*. The kit for OI test contained sixteen pens already impregnated with sixteen familiar different odours. Each was placed in front of the nostrils of each participant at different times for about 3 seconds and asked to select the source substance that matched the presented odour from four different items in a forced choice procedure (four-alternative forced choice). A period of about 30 seconds was allowed before another pen was presented to prevent adaptation or olfactory desensitization [19, 20]. The number of correctly selected source substances by the participant was recorded. The minimum score that a participant can get is zero and the maximum is 16 [21].

*Odour Discrimination Testing*. The test kit for OD contains forty-eight pens which were arranged in sixteen triplets. In each triplet, two of the pens contained the same odour while the third contained another odour. Each pen in a triplet was presented to the nostril of each participant and was expected to identify the pen with a different odour (three-alternative forced choice) [19–21]. They were only allowed to sample each odour once. The triplets were presented at intervals of at least 30 seconds and the individual odour pens at intervals of approximately 3 seconds. When the participant correctly identified the pen with a different odour she was given a point score and when she got it wrong, she had score of zero. The process was repeated for all the 16 triplet pens. The minimum score that a participant can get is zero and the maximum is 16 [21].

*Odour Threshold Testing*. The test kit for OT contains forty-eight pens which were arranged in sixteen triplets. In each triplet, two of the pens were without odour, while the third pen was impregnated with different concentrations of n-butanol solution. The lowest concentration of n-butanol was 4%. The test process started with the triplet containing the pen with the lowest concentration of n-butanol solution and then moved stepwise up (i.e., lowest to highest concentrations) until the participant identified the pen with a different smell in a triplet [19, 20]. After the correct recognition of the pen with n-butanol odour in a triplet, the pens were shuffled and again presented to the participant in a random order. If she was able to recognize the pen with n-butanol odour in a triplet in the second time, a reversal of the staircase was commenced. She was then presented with the triplets in a reversed staircase (i.e., highest to lowest concentrations) until she could no longer identify the pen with the odour of n-butanol. The staircase was then reversed. The staircase reversal was repeated until the seventh time. The triplets were presented at 30-second intervals. The threshold is the mean of the last 4 of 7 staircase reversal points. Thus, the minimum score that a participant can get is one and the maximum is 16 [21].

The sum of OT, OD, and OI values gave the threshold, discrimination, and identification (TDI) score which can range from 1 to 48 in an individual. TDI score ≤15 was considered as functional anosmia and TDI score of 16 to 30 was considered as hyposmia [22]. For women with ages 16 to 35 years, TDI scores of ≤30.5, OT scores of ≤6.5, OD scores of ≤10, and OI scores of ≤11 were suggestive of hyposmia. For those with ages 36 to 55 years, TDI scores of ≤28.8, OT scores of ≤5.5, OD scores of ≤10, and OI scores of ≤11 were suggestive of hyposmia. Any value above this will suggest normosmia [23].

#### 2.3.6. Test of Taste

Sixteen filter paper strips (“taste strips,” Burghart, Wedel, Germany) impregnated at one end with 0.05, 0.1, 0.2, or 0.4 g/mL of sucrose (sweet taste); 0.05, 0.09, 0.165, or 0.3 g/mL of citric acid (sour taste); 0.016, 0.04, 0.1, or 0.25 g/mL of sodium chloride (salty taste); or 0.0004, 0.0009, 0.0024, or 0.006 g/mL of quinine hydrochloride (bitter taste) were used to test the gustatory function of the participants [24, 25]. The taste strips were applied on the left and right side of the protruded tongue, 1.5 cm from the tip, at different times resulting in a total of 32 trials.

Before the test of taste was performed, participants were given clean water to rinse their mouth. The taste strips were then presented in increasing concentrations, alternating the side of presentation. The taste strips were presented at intervals of 30 seconds. The participants were told to identify the taste, with their tongue still protruded, from a list of four descriptors, that is, sweet, sour, salty, and bitter (four-alternative forced choice). Wrong identification of each taste strip was given score of 0 and correct identification was given score from 1 to 4. Correct identification of the taste at the lowest concentration was 4 and at the highest one was 1. The minimum and maximum score on one side of the tongue ranged from 0 to 16. Thus, the Total Taste Strip (TTS) score, which is the sum of the score from both sides of the tongue, ranged from 0 to 32. Sweet score < 2, sour score < 2, salty score < 2, bitter score < 1 for each side of the tongue, total score < 9 on each side of the tongue, and TTS < 19 were suggestive of hypogeusia [23].

### 2.4. Statistical/Data Analysis

The qualitative sociodemographic characteristics of the HIV infected and uninfected women were compared using Chi-square tests. The comparison of mean subjective smell and taste scores between HIV infected and uninfected adults was done using the independent samples *t*-test. Descriptive data were presented as mean ± standard deviation. Chi-square or Fisher's exact test was used where appropriate to test the association. Test of associations for categorical variables was done using Chi-square/Fisher's exact test. Test of association for quantitative variables was done using Student's *t*-test and ANOVA. The associations between TDI and TTS of the HIV infected adults and their RNA viral load, CD4 cell count level, and HAART were tested with Pearson correlation. Level of significance was determined at *p* < 0.05, two-tailed level at 95% Confidence Interval (CI), and correlation coefficient (*r*).

## 3. Results

### 3.1. Sociodemography

One hundred and sixty-two women participated in the study comprising 83 HIV infected women and 79 HIV uninfected women. The age of the HIV infected women ranged from 20 to 70 years, mean 43.67 years ± 10.72, and that of HIV uninfected women ranged from 20 to 65 years, mean 41.48 years ± 10.99. The mean duration of HIV diagnosis was 80.43 months ± 40.05. All the HIV infected women were on HAART for a mean duration of 69.14 ± 38.92 months. Thirty-eight HIV (45.8%) infected women were on combination therapy with nevirapine, zidovudine, and lamivudine; 35 (42.2%) were on combination of tenofovir, lamivudine, and efavirenz; five (6.0%) were on combination of tenofovir, lamivudine, zidovudine, and atazanavir; and another five (6.0%) women were on combination therapy of tenofovir, lamivudine, lopinavir, and ritonavir. The sociodemographic characteristics of the participants are shown in Table 1.

### 3.2. Virology and Immunology

The Nadir CD4 cell count of the HIV infected women was from 8 to 527 cells/*μ*L, mean of 186.44 cells/*μ*L ± 117.25, while the most recent CD4 cell count was from 34 to 2892 cells/*μ*L, mean of 548 cells/*μ*L ± 397.79. In addition, the Nadir viral load was from 257 to 1542776 copies/mL, mean of 215385.73 copies/mL ± 309577.61. The most recent viral load level in 57 (68.7%) HIV infected women was undetectable (<200 copies/mL). The remaining 27 HIV infected women had viral load ranging from 244 to 66918 copies/mL, mean of 3002.16 copies/mL ± 10792.93.

### 3.3. Subjective Self-Rating of Smell and Taste Function of HIV Infected and Uninfected Women

On a visual analogue scale (VAS) from 0 to 10, HIV infected women rated their smell function to be from 1 to 10, with mean of 8.8 ± 1.4, and their taste function to be from 2 to 10, with mean of 9.1 ± 1.2. The HIV uninfected women rated their smell function to be from 5 to 10, with mean of 9.1 ± 1.2, and their taste function to be from 2 to 10, with mean of 8.6 ± 1.9. There was no significant difference in the subjective self-rating of smell (*p* = 0.67) and taste (*p* = 0.84) between the two groups.

### 3.4. Objective Assessment of Smell and Taste Function of HIV Infected and Uninfected Women

The mean smell and taste scores of both the HIV infected and uninfected women are shown in Table 2. The mean scores of all the components of both smell and taste tests were greater in the HIV uninfected women than in the HIV infected women. There was a significant difference between mean TDI scores of HIV infected women and HIV uninfected women (*p* = 0.000). There was also a significant difference in the mean TTS score of HIV infected women and HIV uninfected women (*p* = 0.040).

Hyposmia is seen in 33 (39.8%) HIV infected women and 10 (12.7%) HIV uninfected women (*p* = 0.0002) as shown in Table 3. The proportion of participants with hyposmia and the type of olfactory tests is shown in Table 4. Similarly, hypogeusia was seen in 13 (15.7%) HIV infected women and 1 (1.3%) HIV uninfected woman (*p* = 0.0110; OR = 14.4857 (1.8473–113.5894)) as shown in Table 3. There was a significant correlation between HIV disease stage and hyposmia (*r* = 0.873; *p* = 0.000) and hypogeusia (*r* = 0.851; *p* = 0.021). In addition, as the stage of HIV disease increases, the proportion of HIV infected women with hyposmia and hypogeusia increases (Table 5). Only 6 (7.2%) HIV infected women have both hyposmia and hypogeusia.

### 3.5. Comparing Subjective Self-Reporting Assessment with the Objective Assessment of Smell and Taste Function of HIV Infected Women

There was a significant difference between the subjective self-reporting and objective smell (TDI) assessment of the HIV infected women (*p* = 0.048). However, there was no significant difference between self-reporting and objective taste (TTS) assessment of the HIV infected women (*p* = 0.064).

The type of HAART regimen and the proportion of HIV infected participants with hyposmia and hypogeusia are shown in Table 6. Two HIV infected women in this study were not on HAART.

**Figure 1:**
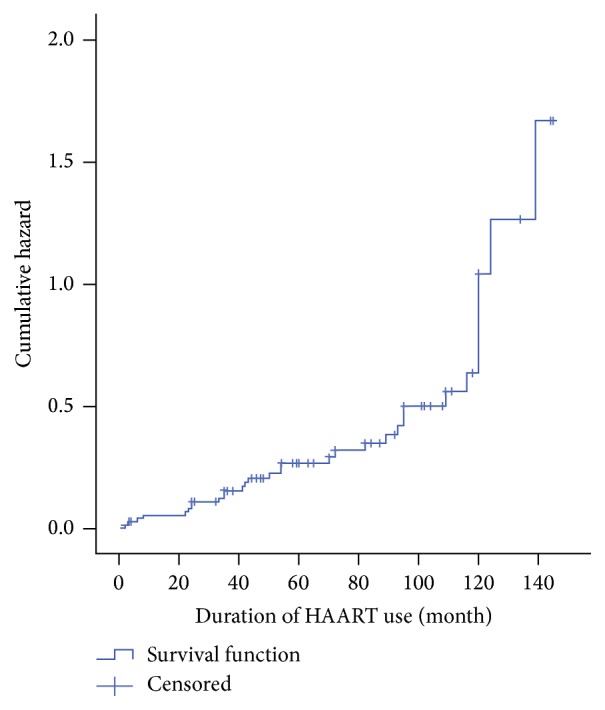
Hazard function of HAART on HIV infected participants with hyposmia.

Those on HAART have significantly higher odds of hyposmia compared with the controls (*p* = 0.000; OR = 2.13 (1.597–2.828)). The odds of hyposmia increased with increased duration of HAART usage (Figure 1).

**Figure 2:**
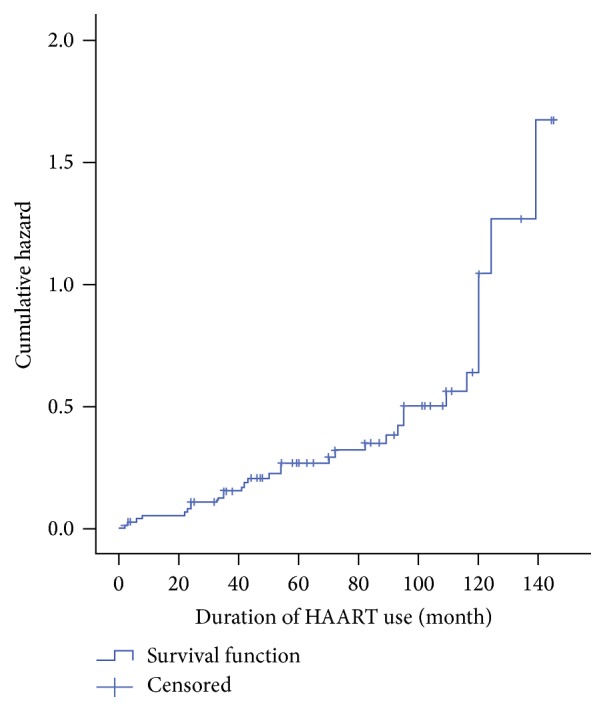
Hazard function of HAART on HIV infected participants with hypogeusia.

Similarly, those on HAART have significantly higher odds of hypogeusia compared with the controls (*p* = 0.001; OR = 15.29 (1.950–119.99)). The odds of hypogeusia increased with increased duration of HAART usage (Figure 2).

## 4. Discussion

The cause of smell and taste dysfunction is multifactorial, even in women. The contribution of HIV infection and antiretroviral medications was investigated in this study using subjective assessment based on visual analogue scale scores and validated objective smell and taste tests. To the best of our knowledge, this is the first study which used these methods to investigate the smell and taste of HIV infected and uninfected women in Sub-Sahara Africa.

Little attention is given to investigating smell and taste function in HIV infected women. Most people with smell dysfunction may not recognize it as evidenced by the significant difference between the subjective self-reporting and objective olfactory assessment of the women in this study. Although none of the participants in this study had anosmia, there was a trend towards more hyposmia/anosmia among HIV infected women than in the HIV uninfected women (Table 4). The prevalence of hyposmia was higher in HIV infected women (39.8%) than in HIV uninfected women (12.6%). The higher prevalence of hyposmia in the HIV infected women may be due to inflammatory changes which occurred in the olfactory mucosa from readily encountered opportunistic infection of the upper respiratory tract and the neurotropic effect of HIV on the olfactory nerve [10, 12, 13]. Chronic inflammation, which is a common disease among the HIV infected individuals, has been implicated in the aetiology of hyposmia [13]. Although none of the participants had rhinosinusitis at the time of investigation, the repeated inflammation of the olfactory mucosa from recurrent rhinosinusitis over a long period of time can damage the olfactory epithelium with resultant smell dysfunction. The improved immunity of the HIV infected women as evidenced by a higher level of mean latest CD4 cell count compared to the Nadir CD4 cell count would have reduced the possibility of opportunistic rhinosinusitis in them.

The prevalence of hypogeusia in HIV infected women (15.7%) was higher than in healthy HIV uninfected women (1.3%). Most people with taste dysfunction may notice and report it as evidenced by the nonsignificant difference between the subjective and objective taste assessment of the women in this study. HIV infection predisposes people to oral candidiasis and lesions which may cause taste dysfunction [12]. Dryness of the oral cavity from inadequate fluid intake and inflammation of salivary glands can also contribute to gustatory dysfunction by reducing transportation of taste chemicals to taste buds. Although whole mouth taste dysfunction is rare, taste sensitivity is directly related to the number of taste papillae and taste buds that are stimulated [26, 27]. Ageing process and viral infections like HIV can reduce the quantity of taste buds and papillae in the upper orodigestive tract [28, 29]. These chemosensory abnormalities can impair food intake thereby contributing to significant weight loss [14]. HIV associated wasting is an increasingly common clinical manifestation of AIDS. Although rarely appreciated, taste dysfunction can alter choice of food and pattern of its consumption thereby predisposing to weight loss or gain, hypertension (because of frequent consumption of salt in bigger quantity to improve food taste), and diabetes (because of frequent sugar consumption to improve food taste) and, in some cases, may impair immunity and even cause death. The increased preferences for salty and sweet tasting substances in 39.8% and 33.7% of HIV infected women (Table 1) will encourage consumption of more than needed salt (electrolytes) and sugar. In individuals who are at risk of developing hypertension, excess salt intake will cause expansion of body fluid volume resulting in hypertension. In addition, excess sugar consumption may increase the risk of excessive weight gain or diabetes in those who are prone to it [30, 31]. The comorbid diseases of hypertension and diabetes in HIV infected women will definitely impair their quality of life and standard of living.

Women require optimal nutrient intake for maintenance of their body function, menstruation, and proper growth and development of pregnancy. HIV infected women require good nutrition for improved immunologic status to fight against opportunistic infections. Poor nutrition may worsen HIV disease progression, depress immune system with resultant frequent opportunistic infections, and impair optimal response to antiretroviral medication. The perceptions of taste and smell play important roles in stimulating caloric intake and optimising nutrient absorption through cephalic phase reflexes [2–4]. In instances where nutrient intake is inadequate and chemosensory perception is considered a likely contributor to taste and smell losses, the use of supplements and flavor enhancers like monosodium glutamate may improve both quality and quantity of intake [3, 32].

Reports have shown that OT can reflect more on the function of the peripheral olfactory system (perception of odour) while tests of OI and OD reflect more on the cognitive performance (recognition and memory of specific odour) [33, 34]. Majority of HIV infected and uninfected women with hyposmia will have problem with odour perception while lesser proportion will have difficulty in recognizing or keeping the memory of the specific odour. These significant taste and smell losses in HIV infected women may be of clinical significance in the development or progression of HIV associated wasting [35]. It is therefore important to make them aware of this problem and warn them of the associated risk. A significant correlation was established between Centers for Disease Control and Prevention stage of HIV infection and smell and taste function. CD4-receptors containing cells are the portal of entry of HIV, and the rate of CD4 cell destruction is directly proportional to progressive replication of HIV and susceptibility to opportunistic infections. Administration of HAART will improve the CD4 cell counts level, lower HIV disease stage, and then improve the hyposmia and quality of life. Losses of smell and taste have been reported to be rare side effects of some classes of medication [15, 16, 36]. The effects of HAART on smell and taste function of HIV infected individuals have been documented [37–39]. In this study, the HIV infected women on HAART are 2.13 and 15.29 times more likely to develop hyposmia and hypogeusia, respectively, than the controls. It would have been good to see this relationship between HIV infected women on HAART and those not on HAART. The analysis could not be done because only two HIV infected women in this study were not on HAART. This needs to be investigated in the future. This present study also showed that the longer the use of HAART, the higher the odds of hyposmia and hypogeusia. The long mean duration of HAART use by HIV infected women might have contributed to the observed increase in the mean CD4 cell count and decrease in mean RNA viral load which are indicators of treatment outcome and prognosis. It is also a long enough time for dysfunctioning taste buds and papillae to have regenerated thereby contributing to the observed low proportion of subjects with hyposmia in this study.

## 5. Conclusions

Hyposmia and hypogeusia are commoner among the HIV infected women than the HIV uninfected women and the risk increases with an increased duration of highly active antiretroviral therapy.

## Figures and Tables

**Table 1:** Sociodemographic characteristics of the participants.

Variable	HIV infected women	HIV uninfected women
Age (years)		
35 and below	20 (24.1)	56 (70.9)
36–55	51 (61.4)	18 (22.8)
56 and above	12 (14.5)	5 (6.3)
Marital status		
Single	6 (7.2)	31 (39.2)
Married	64 (77.1)	47 (59.5)
Divorced	3 (3.6)	0
Widow	10 (12.0)	1 (1.3)
Occupation		
Class 1	6 (7.2)	12 (15.2)
Class 2	26 (31.3)	24 (30.4)
Class 3	14 (16.9)	3 (3.8)
Class 4	37 (44.6)	32 (40.5)
Class 5	0	8 (10.1)
Preference for taste		
Salt	33 (39.8)	29 (36.8)
Sweet	28 (33.7)	34 (43.0)
Sour	14 (16.9)	11 (13.9)
Bitter	8 (9.6)	5 (6.3)

**Table 2:** Comparison of smell and taste of the participants.

Variable	Mean ± SD	Mean ± SD	Level of significance (*p* value)
	HIV infected women	HIV uninfected women	
Smell			
Olfactory threshold	8.1 ± 5.0	11.4 ± 2.1	0.000
Olfactory discrimination	10.1 ± 5.0	11.7 ± 2.6	0.012
Olfactory identification	9.0 ± 4.7	13.3 ± 3.0	0.000
TDI	27.3 ± 11.7	36.3 ± 5.0	0.000
Taste			
Sweet	3.3 ± 0.9	3.8 ± 0.6	0.000
Sour	3.1 ± 0.9	3.3 ± 0.9	0.034
Salty	3.1 ± 1.1	3.5 ± 0.8	0.000
Bitter	3.2 ± 1.0	3.7 ± 0.6	0.000
TTS	25.3 ± 5.9	29.0 ± 3.8	0.040
Subjective smell	8.8 ± 1.4	9.1 ± 1.2	0.67
Subjective taste	7.6 ± 2.9	8.6 ± 1.9	0.84

**Table 3:** Participants with hyposmia and hypogeusia.

Variables	TDI		TTS	
	Hyposmia	Normosmia	Hypogeusia	Normogeusia
	*n* (%)	*n* (%)	*n* (%)	*n* (%)
HIV infected women				
35 and below	8 (9.6)	12 (14.5)	4 (4.8)	16 (19.3)
36–55	19 (22.8)	32 (38.6)	6 (7.2)	45 (54.2)
56 and above	6 (7.2)	6 (7.2)	3 (3.6)	9 (10.8)
Total	**33 (39.8)**	**50 (60.2)**	**13 (15.7)**	**70 (84.3)**
HIV uninfected women				
35 and below	5 (6.3)	51 (64.6)	0	56 (70.9)
36–55	3 (3.8)	15 (19.0)	1 (1.3)	17 (21.5)
56 and above	2 (2.5)	3 (3.8)	0	5 (6.3)
Total	**10 (12.7)**	**69 (87.3)**	**1 (1.3)**	**78 (98.7)**

**Table 4:** Types of olfactory dysfunction in the participants.

Olfactory tests	Hyposmia *n* (%)		Odds ratio	95% CI	*Z* statistic	*P* value
	HIV infected women	HIV uninfected women				
OT	27 (81.8)	6 (60.0)	5.8661	2.2671–15.1781	3.648	0.0003
OD	15 (45.5)	4 (40.0)	4.1360	1.3086–13.0722	2.418	0.0156
OI	6 (18.2)	2 (20.0)	3.0000	0.5871–15.3302	1.320	0.1868
TDI	33 (100.0)	10 (100.0)	4.5540	2.0551–10.0913	3.734	0.0002

**Table 5:** Relationship of HIV infection stage with hyposmia and hypogeusia.

Stage	Nadir CD4^+^ cell count (cells/*μ*L)	Olfaction of HIV infected women		Gustation of HIV infected women	
		Hyposmia	Normosmia	Hypogeusia	Normogeusia
1	≥500	8 (9.6%)	15 (18.1%)	3 (3.6%)	7 (8.4%)
2	200–499	11 (13.2%)	16 (19.3%)	5 (6.0%)	27 (32.5%)
3	<200	14 (16.9%)	19 (22.9%)	5 (6.0%)	36 (43.4%)
		**33 (39.7)**	**50 (60.3)**	**13 (15.7)**	**70 (84.3)**

**Table 6:** Relationship of highly active antiretroviral therapy with hyposmia and hypogeusia.

Highly active antiretroviral therapy (HAART)	Olfaction test (TDI)		Gustation test (TTS)	
	Hyposmia	Normosmia	Hypogeusia	Normogeusia
Nevirapine, zidovudine, and lamivudine	**16**	**20**	**7**	**29**
Tenofovir, lamivudine, and efavirenz	**13**	**22**	**5**	**30**
Tenofovir, lamivudine, zidovudine, and atazanavir	**1**	**4**	**1**	**4**
Tenofovir, lamivudine, lopinavir, and ritonavir	**2**	**3**	**0**	**5**
